# High-risk pathogenic germline variants in blood relatives of *BRCA1/2* negative probands

**DOI:** 10.1007/s12282-024-01615-0

**Published:** 2024-07-13

**Authors:** Reiko Yoshida, Tomoko Kaneyasu, Arisa Ueki, Hideko Yamauchi, Shozo Ohsumi, Shinji Ohno, Daisuke Aoki, Shinichi Baba, Junko Kawano, Naomichi Matsumoto, Masao Nagasaki, Takayuki Ueno, Hitoshi Inari, Yusuke Kobayashi, Junko Takei, Osamu Gotoh, Mitsuyo Nishi, Miki Okamura, Keika Kaneko, Megumi Okawa, Misato Suzuki, Sayuri Amino, Mayuko Inuzuka, Tetsuo Noda, Seiichi Mori, Seigo Nakamura

**Affiliations:** 1 Division of Cancer Genomics, Cancer Institute, Japanese Foundation for Cancer Research (JFCR), 3-8-31 Ariake, Koto-ku, Tokyo, Japan; 2 Institute for Clinical Genetics and Genomics, Showa University, 1-5-8 Hatanodai, Shinagawa-ku, Japan; 3 Project for Development of Innovative Research on Cancer Therapeutics, Cancer Precision Medicine Center, Japanese Foundation for Cancer Research, Tokyo, 135-8550 Japan; 4 Department of Clinical Genetic Oncology, Cancer Institute Hospital, JFCR, 3-8-31 Ariake, Koto-ku, Tokyo, Japan; 5 Department of Breast Surgical Oncology, St. Luke’s International Hospital, 10-1 Akashi-cho, Chuo-ku, Tokyo, Japan; 6 National Hospital Organization Shikoku Cancer Center, 160 Kou, Minamiumemoto-machi, Matsuyama, Ehime Japan; 7 Breast Oncology Center, Cancer Institute Hospital, Japanese Foundation for Cancer Research, 3-8-31 Ariake, Koto-ku, Tokyo, Japan; 8 Department of Obstetrics and Gynecology, Keio University School of Medicine, 35 Shinano-machi, Shinjuku-ku, Tokyo, Japan; 9 Sagara Hospital, 3-31 Matsubara-cho, Kagoshima, Japan; 10 Department of Human Genetics, Yokohama City University Graduate School of Medicine, Fukuura 3-9, Kanazawa-ku, Yokohama, Japan; 11 Department of Biomedical Information Analysis, Center for Genomic Medicine, Graduate School of Medicine, Kyoto University, 53 Shogoinkawahara-cho, Sakyo-ku, Kyoto, Japan; 12 Division of Breast Surgical Oncology, Showa University School of Medicine, 1-5-8 Hatanodai, Shinagawa-ku, Tokyo, Japan; 13 Cancer Institute, JFCR, 3-8-31 Ariake, Koto-ku, Tokyo, Japan; 14 Department of Genetic Diagnosis, Cancer Institute Hospital, JFCR, 3-8-31 Ariake, Koto-ku, Tokyo, Japan

**Keywords:** Cascade testing, Blood relative analysis, Genetic testing, Hereditary breast and ovarian cancer syndrome, Pathogenic germline variant

## Abstract

**Background:**

Tailored, preventive cancer care requires the identification of pathogenic germline variants (PGVs) among potentially at-risk blood relatives (BRs). Cascade testing is carried out for BRs of probands who are positive for PGVs of an inherited cancer but not for negative probands. This study was conducted to examine the prevalence of PGVs for BRs of PGV-negative probands.

**Methods:**

PGV prevalence was assessed for 682 BRs of 281 probands with *BRCA1/BRCA2* wild-type hereditary breast and ovarian cancer (HBOC) syndrome.

**Results:**

PGVs were discovered in 22 (45.8%) of the 48 BRs of the PGV-positive probands and in 14 (2.2%) of 634 BRs of the PGV-negative probands. Eleven PGVs on high-risk *BRCA1*, *BRCA2*, and *TP53* genes were present only in BRs and not in the probands (probands vs BRs in Fisher exact test; *p* = 0.0104; odds ratio [OR] = 0.000 [0.000–0.5489 of 95% confidence interval]), partly due to the nature of the selection criteria. The enrichment of high-risk PGVs among BRs was also significant as compared with a non-cancer East Asian population (*p* = 0.0016; OR = 3.0791 [1.5521–5.6694]). PGV prevalence, risk class of gene, and genotype concordance were unaffected by the cancer history among BRs.

**Conclusion:**

These findings imply the necessity to construct a novel testing scheme to complement cascade testing.

**Supplementary Information:**

The online version contains supplementary material available at 10.1007/s12282-024-01615-0.

## Introduction

Advancements in our understanding of the genetic risk of inherited cancer have revolutionized preventive health-care regimes for pre-symptomatic individuals, particularly in terms of surveillance and risk-reducing surgery [1, 2]. Tailored, preventive care requires the efficient and accurate identification of pathogenic germline variants (PGVs) among potentially at-risk blood relatives (BRs). To date, cascade testing has been conducted as a general practice to identify heritable PGVs among BRs of positive probands, and this has reduced the costs associated with screening and the need for additional testing among relatives [3–5]. Cascade testing was traditionally conducted using Sanger sequencing of the specific variant at a single site; this has evolved in recent years, and now identifies genetic risk through comprehensive next-generation sequencing of multi-gene panels [3–5].

Cascade testing is based on the premise that inherited cancer represents an autosomal dominant trait with sufficient penetrance [3]. However, in reality, inherited cancer can exhibit as a spectrum of variability in terms of penetrance, and is highly dependent on the PGV as well as the risk class of a number of causative genes [4, 5]. This is the case for hereditary breast and ovarian cancer (HBOC) syndrome, which is defined as an inherited cancer-susceptibility syndrome with multiple blood relatives having breast and/or ovarian cancer. Indeed, multi-gene panel testing has identified numerous [6] and discrete [4, 7, 8] PGVs among probands and other individuals with suspected HBOC.

Despite relatively low rates of uptake of cascade testing in the past, recent years have seen continued reasonable success in the identification of unrecognized PGV carriers, which has popularized the testing regime [3–5]. Cascade testing, however, relies on the proband being positive for a PGV, and, as such, the prevalence of PGVs among BRs of PGV-negative probands has not yet been investigated.

In the current study, we comprehensively assessed 30 breast cancer-susceptibility genes for 682 BRs, 2 spouses, and 281 probands with HBOC syndrome and a strong family history of the disease. In an effort to identify PGVs on other HBOC syndrome causative genes, all selected probands were wildtype for *BRCA1*/*BRCA2* alleles. Our findings imply the necessity to develop a genetic testing scheme for the BRs of PGV-negative probands.

## Methods

### Study overview

In the current study, HBOC syndrome is defined as an inherited cancer-susceptibility syndrome wherein multiple BRs have breast cancer, ovarian cancer, or both. Patients with HBOC syndrome and their BRs were selected from six academic and cancer hospitals in Japan: Showa University Hospital (Hatanodai, Tokyo), Cancer Institute Hospital (Ariake, Tokyo), St. Luke’s International Hospital (Akashi-cho, Tokyo), Shikoku Cancer Center (Minamiumemoto-cho, Matsuyama), Sagara Hospital (Matsubara-cho, Kagoshima), and Keio University Hospital (Shinano-machi, Tokyo). These institutions participated in the “Project for Development of Innovative Research on Cancer Therapeutics” (P-DIRECT; 2014–2015) research program, and in the succeeding “Project for Cancer Research and Therapeutic Evolution” (P-CREATE; 2016–2022) program, granted by the Japan Agency for Medical Research and Development (AMED). Research subjects were *BRCA1/BRCA2* mutation-negative HBOC patients (all probands had breast and/or ovarian cancer) and their BRs and spouses. Part of this analysis was reported previously [7].

### Eligibility criteria of the probands

In the current study, individuals with a diagnosis of breast or ovarian cancer were included in the study if they met any of the following additional criteria: (1) two or more first-degree BRs suffered from breast or ovarian cancer; or (2) one or more first-degree BRs suffered from breast or ovarian cancer that: (2-a) was diagnosed before the age of 40 years, (2-b) arose as a part of synchronous or metachronous bilateral primary breast cancer, and/or (2-c) arose as a part of synchronous or metachronous multiple primary cancer; or (3) one or more first- or second-degree female BRs suffered from breast or ovarian cancer, or one or more first- or second-degree male BRs suffered from prostate cancer [7]. Cancer family histories (up to third-degree BRs for 279 families and up-to fourth-degree BRs for 2 families) were taken by genetic counselors. The proband ID is also used as the family ID.

### Eligibility criteria, contact method, and genetic counseling of the blood relatives and spouses

**Fig. 1:**
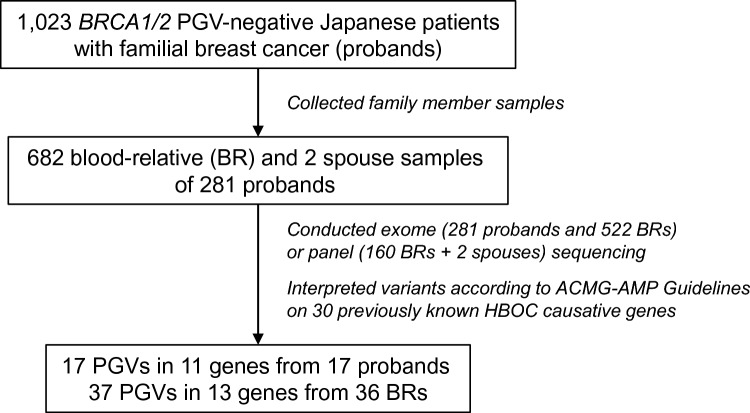
Study design. We first sequenced the exomes of 1,023 *BRCA1*/*BRCA2* wild-type Japanese patients with familial breast cancer (probands). Samples from 684 family members (682 BRs and 2 spouses) of 281 probands, selected from availability of samples, were subsequently rendered to exome or target-panel sequencing. We analyzed prevalence and risk class of mutated genes of the probands and BRs focusing on 30 HBOC-causative genes. BR, blood relative; PGV, pathogenic germline variant; ACMG, the American College of Medical Genetics and Genomics; AMP, the Association of Molecular Pathology; and HBOC, hereditary breast and ovarian cancer

We describe the genetic findings from 281 cases and their 684 family members (682 BRs and 2 spouses), all of whom were selected based on the sample and consent availability (Fig. 1). BRs or spouses were first contacted by the proband and asked to participate in the study. Where the BR or spouse agreed, a packet containing a printed document of informed consent and a saliva collection kit were mailed to the BR or spouse, and the saliva was returned with a signed consent form to the Cancer Precision Medicine Center of Japanese Foundation for Cancer Research. Alternatively, BRs and spouses directly visited the clinic of one of the participating hospitals; albeit, few chose this avenue for participation. For those who visited the clinic, genetic counseling was provided to all probands, BRs, and spouses prior to participation. Genetic counseling was provided to all other BRs and spouses who participated in the study through mail when the result was provided to them at the clinic. Pedigree charts were used to obtain characteristics of all family members at each institution.

### *BRCA1/2* mutation test and sample acquisition

Germline genetic testing to confirm *BRCA1/BRCA2* wildtype for each of the probands was conducted at FALCO Biosystems (Shimizu-cho, Kyoto; 2014–2016) or at Ambry Genetics (Aliso Viejo, California; using OvaNext or CustomNext 25-gene panel; 2017–2022). All *BRCA1*/*BRCA2* negative probands received exome sequencing [7] and were examined for the presence of PGVs across 30 HBOC-causative genes (see Supplementary Methods). Blood was collected from probands, and saliva or blood was provided by BRs/spouses through mail or direct visit.

### Statistical analyses

Mann–Whitney *U*-test and Fisher’s exact test were performed using GraphPad Prism (ver. 9.4.1) or R (ver. 4.2.1) software.

Sections of “Subject overlap between previous and current studies”, “Sample preparation for sequencing”, “Library preparation and target-panel or exome sequencing”, “Gene selection and risk assignment”, “Germline variant analysis”, and “Population databases and disease variant databases” appear in the Supplementary Methods.

## Results

### Study cohort

We previously reported the prevalence of germline variants among Japanese patients with *BRCA1*/*BRCA2*-wildtype HBOC syndrome and a strong family history [7]. In that study, we noted that the PGV-carrying status of HBOC-causative genes was complex between index cases (*n* = 13) and their BRs (*n* = 34), as well as between affected and unaffected BRs [7]. The present study was undertaken to better understand this complexity. We expanded the cohort to 684 family members (682 BRs and 2 spouses) of 281 index cases, of which germline samples were obtained, regardless of the proband genotype (Fig. 1 and Table S1A). Degree of relatedness and gender were significantly associated with the rate of subject participation (Table S1B).

We conducted exome sequencing for samples from all 281 probands and 522 BRs. The remaining samples from 160 BRs and 2 spouses were sequenced with target panels covering all exons of 30 previously known HBOC-causative genes (Fig. 1 and Table S2; target genes are described in the Supplementary Methods). The clinical features of the BRs and spouses who received genetic analyses are described in Table 1.

**Table 1:** Characteristics of blood relatives (BRs) and spouses who received a genetic analysis

Category		No. of subjects with PGV	No. of subjects without PGV	Fisher’s exact test *P* value
*Degree of relation*				
	1st	27^a^	470	0.8491
	2nd	7	112	0.658
	3rd	2	62	0.5658
	4th	0	2	1
	Spouse	0	2	1
*Gender*				
	Male	9	181	0.8489
	Female	27^a^	467	0.8489
*Affected cancer type*				
	BC	11	140	0.2169
	OC	0	5	1
	BC + OC	0	1	1
	BC + other HR-related cancer^b^	0	1	1
	BC + other cancer	1	7	0.3526
	OC + other cancer	0	3	1
	HRD-related cancer^b^ other than BC + OC	1	9	0.4198
	Other cancer^c^	3^a^	25	0.1778
	Unaffected	20	457	0.0636
Total		36	648	

BR; blood relative, PGV; pathogenic germline variant, BC; breast cancer, OC; ovarian cancer and HRD; homologous recombination deficiency

^a^One blood relative had two PGVs

^b^Breast, ovarian, pancreatic, and prostate cancers were considered as HRD-related cancer [1]

^c^Other cancers were melanoma, colorectal cancer, gastric cancer, cervical cancer, uterine cancer, lung cancer, leukemia, renal cancer, malignant lymphoma, head and neck cancer, and brain tumor and bladder cancer

### Detection of PGVs in BRs of PGV-negative probands

Variant interpretations were used to ascertain PGVs on probands and BRs/spouses. Overall, 17 PGVs (11 genes) and 37 PGVs (13 genes) were identified for 17 probands and 36 BRs, respectively (Fig. 1 and Table S3). Two spouses were negative for PGV. Representative pedigree charts with PGV information are shown in Figures S1 and S2, with additional information provided in the Supplementary Document. Many families exhibited complexity in concordance with PGV-carrying status between probands and BRs, and between affected and unaffected BRs, as noted previously [7].

**Fig. 2:**
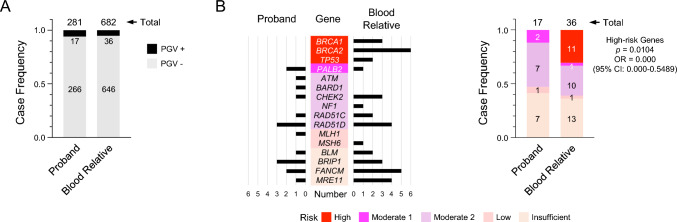
Differences in mutated genes between probands and BRs. **A** Frequency of PGV carriers among probands and BRs. Numbers of PGVs are shown. **B** Prevalence of risk class of mutated genes between probands and BRs. Shown are the number of PGVs per gene for probands and BRs (left panel) and the frequency of risk class for probands and BRs (right panel). Frequency is based on the number of cases. Note that all 281 probands had wild-type *BRCA1/2* alleles due to the study design. The *p* value was computed using Fisher’s exact test for PGV carriers of high-risk genes. BR, blood relatives; PGV, pathogenic germline variant; OR, odds ratio; and 95% CI; 95% confidence interval

The prevalence of PGVs on HBOC-causative genes was similar between probands and BRs (6.0% and 5.3%; Fig. 2A). However, in some cases, PGVs found for BRs were absent in the probands (Fig. 2B, left panel). *BRCA1*/*BRCA2* wild-type patients were selected as the index cases; therefore, all probands were negative for PGVs of *BRCA1*/*BRCA2* genes (Fig. 2B, proband in left panel) [7]. However, *BRCA1* and *BRCA2* PGV carriers were identified among BRs (Fig. 2B, BRs in left panel). Similarly, we detected mutations in *TP53*, *NF1*, and *MSH6* genes in the BRs, which were also absent among the probands (Fig. 2B left panel). After stratifying the mutated genes in terms of risk (see also “Gene selection and risk assignment” in Supplementary Methods), we found a significant difference in the presence of PGVs within high-risk genes in BRs as compared with probands (Fisher’s exact test, *p* = 0.0104; odds ratio [OR] = 0.000 [95% confidence interval, 0.000–0.5489]; Fig. 2B right panel). This significant enrichment was further confirmed against the metadata from a non-cancer East-Asian population (Tables S4 and S5).

### Discordance of PGVs between probands and BRs

**Fig. 3:**
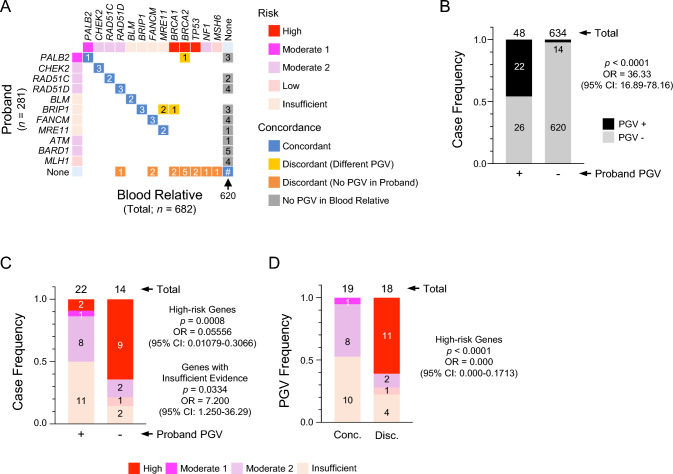
Risk class of mutated genes and concordance of PGV detection in BRs. **A** Concordance of PGVs between probands and BRs. The number in the square indicates the specific BR with concordant or discordant detection of PGVs. Concordance is defined by the same PGV detection between the proband and BR; discordance is defined based on either the detection of a different PGV between the proband and BR, or detection of a PGV in the BR but not in the proband. **B** PGVs in the BRs of probands with or without PGVs. Frequency of cases is indicated. The *p* value was computed by Fisher exact test. **C** Frequency of risk class of mutated genes detected in the BRs of probands with or without PGV. Frequency is based on the number of cases. The *p* value was computed using Fisher’s exact tests. **D** Frequency of risk class of mutated genes in BRs of concordant (conc.) or discordant (disc.) PGV detection with respect to the proband. Frequency is based on the number of PGVs. BR, blood relative; PGV, pathogenic germline variant; OR, odds ratio; and 95%CI, 95% confidence interval

We examined concordance/discordance rates for the presence of PGVs between probands and BRs for various genes (Fig. 3A; Table S3). Concordance was observed for PGVs in *PALB2*, *CHEK2*, *RAD51C*, *RAD51D*, *BLM*, *BRIP1*, *FANCM*, and *MRE11* among 19 BRs from 14 families (Fig. 3A; Table S3). Two types of discordance were noted: (1) PGVs identified in BRs but absent in the probands, and (2) different PGV(s) identified in the BR(s) from those in the proband. In the first instance, we identified mutations in *BRCA1*, *BRCA2*, *TP53*, *NF1*, *RAD51D*, *MSH6*, or *FANCM* genes in 14 BRs from 11 families but no PGVs in the probands (Fig. 3A). In the second case, 2 BRs from 2 different families (Fig. 3A) had different PGVs from that of their respective probands; this was as described previously [4, 7, 8]. Specifically, in one family, the BR had a discordant PGV, whereas, in the other family, the BR had two PGVs, one concordant and one discordant (Fig. 3A; Table S3; Supplementary Document). Specifically, one PGV on *TP53* (c. 743G>A [p.R248Q]) was identified as a de novo mutation, as confirmed by trio analysis (Figure S2 and Table S3; A0867; daughter of A0350 and A0866). In a different family, another *TP53* variant (c. 713G>A [p.C238Y]) was found (BR, A0815) and assumed to be de novo, because the BR’s mother (A0235; the proband) and sister (A0812) lacked the variant, and her father had no previous history of cancer (nor was he subjected to testing in this instance; Figure S2 and Table S3). Overall, discordant PGVs were not rare among BRs of *BRCA1*/*BRCA2* wild-type HBOC patients.

### Enrichment of high-risk genes in discordant PGVs

BRs of PGV-positive probands contained a higher number of mutations on genes with insufficient evidence of risk (*p* = 0.0334; OR = 7.200 [1.250–36.29]). In contrast, significantly fewer PGVs were found among the BRs of PGV-negative probands than among those of PGV-positive probands (*p* < 0.0001; OR = 36.33 [16.89–78.16]). However, PGVs were still detected in 14 BRs of PGV-negative probands (Fig. 3B), with a significant presence of high-risk genes (*p* = 0.0008; OR = 0.05556 [0.01079–0.3066]) (Fig. 3C). Indeed, by stratifying the BRs in terms of PGV concordance or discordance, we observed enrichment of high-risk genes among the discordant PGVs (Fig. 3D). These findings indicate a clear association between high-risk genes and discordant PGV detection.

### Impact of positive cancer history and route of PGV inheritance

**Fig. 4:**
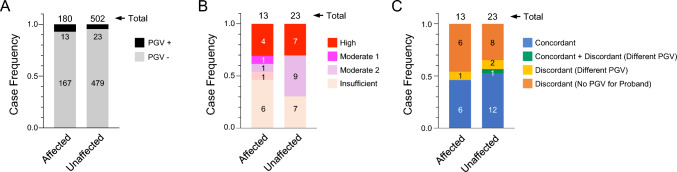
Prevalence, risk class, and concordance of PGVs in BRs with (affected) or without (unaffected) cancer history. **A** Prevalence of PGVs in BRs with (affected) or without (unaffected) cancer history. Frequency is based on the number of cases. **B** Frequency of risk class of mutated genes in BRs with (affected) or without (unaffected) cancer history. Frequency is based on the number of cases. **C** Concordance of PGV detection in BRs with (affected) or without (unaffected) cancer history. Frequency is based on the number of PGVs. BR, blood relative; PGV, pathogenic germline variant

Given that we initially selected index cases with a strong family history of breast or ovarian cancer [7], we considered that there could be enrichment of PGV carriers among BRs with a positive cancer history. However, binary comparisons revealed no significant difference in prevalence (Fig. 4A), risk class (Fig. 4B), or genotype concordance (Fig. 4C) of PGVs between cancer-affected and cancer-unaffected BRs. These observations indicate a negligible impact of positive cancer history on the PGV-carrying status of BRs.

We also considered the relevance of the pattern of PGV inheritance in a family, and sought to determine whether maternal or paternal transmission could be estimated based on the status of breast or ovarian cancer history among BRs (Figure S3). In terms of cancer history, PGVs were estimated as having been inherited maternally and paternally in 16 and 4 families, respectively. For 8 families, insufficient information prohibited an estimation of the route of inheritance (Figure S3). We then compared these estimates with genetic analyses. The genetic analyses revealed maternal and paternal transmissions in 8 and 4 families, indicating that only 6 maternal and 2 paternal transmissions were correctly estimated (Figure S3). In addition, as mentioned above, trio analysis revealed a de novo PGV on *TP53* (Figure S2 and S3). Together, these observations show that it is difficult to estimate the route of PGV inheritance from phenotypic status alone.

## Discussion

The evidence collected to date supports the efficacy of cascade testing as a practical approach for determining PGV-carrying status of BRs of PGV-positive probands. However, it is not uncommon for BRs to have a PGV when the proband is negative [4, 7, 8], and therefore, genetic testing is still recommended in such cases [1]. However, often, the negative result reduces patient concern [1], such that BRs even meeting with the testing criteria are not assessed or subjected to genetic testing. Indeed, the previous clinical studies of hereditary cancer syndrome have focused on BRs of PGV-positive probands using cascade testing [3, 4, 9]; as yet, there has been no study investigating PGV prevalence among asymptomatic BRs of PGV-negative probands with a confirmed cancer diagnosis. Here, we performed comprehensive genetic testing of BRs to identify any PGV from among 30 causative genes associated with HBOC syndrome, regardless of PGV positivity status of the *BRCA1/BRCA2* wild-type proband. As anticipated, 19 BRs (39.6%) had PGVs concordant with their probands, which affirms the method and validates the effectiveness of cascade testing for these BRs.

Noteworthy, we also found that 11 BRs (1.6%) were discordantly positive for PGVs on high-risk *BRCA1*, *BRCA2*, and *TP53* genes, which were absent in the probands. These high-risk genes were significantly enriched in BRs within the current cohort as compared with a non-cancer East-Asian population used as a control. Breast cancer is a common disease that frequently arises without any PGVs and may explain this discordance [10]; for instance, non-hereditary, sporadic breast cancer may coincidently occur for one person, while another BR will exhibit a hereditary form of breast or ovarian cancer. Prevalent and penetrant PGVs, such as those on *BRCA1*/*BRCA2* genes [11, 12], can result in a strong familial incidence. Whereas we only assessed the families of *BRCA1*/*BRCA2* wild-type probands, the selection of families through stringent criteria based on family history [7] as well as the enrichment of first-degree BRs among the study subjects likely led to a prominent bias and a higher prevalence in the identification of PGVs on *BRCA1*/*BRCA2* genes. Additionally, the de novo emergence of PGVs, as observed on the *TP53* allele in two families, should be considered as another source of appearance of a PGV in the family of negative proband.

A personal history of breast, ovarian, or other HBOC-related cancer is regarded as a significant factor among the list of criteria for genetic testing for probands with HBOC syndrome, as is the presence of an affected BR [1, 13]. However, we found no significant difference between affected and unaffected BRs in terms of PGV prevalence, risk class of gene, or genotype concordance. Whereas this may be attributed to the limited number of samples in the cohort, the results may also point to a lower relevance of cancer history among BRs in testing for PGVs. Similarly, whereas PGV inheritance for hereditary cancer syndrome is generally estimated based on the distribution of specific cancer types among BRs on the paternal or maternal side [13], our analysis shows that phenotypic estimations of the route of inheritance are also largely unreliable. Indeed, considering that one of the critical purposes of BR testing is to identify a PGV carrier for preventive medical intervention prior to cancer onset, it may be better to offer genetic testing to all accessible BRs without any selection by phenotype.

There remains some uncertainty in the contribution of PGVs to cancer onset for moderate-risk genes, low-risk genes, and genes with insufficient evidence. The prevalence and impact of PGVs that belong to different risk classes may not be directly compared. To clinically implement genetic testing of BRs of a PGV-negative proband will require rigorous consideration of the ethical aspects of a diagnosis, as well as the cost associated with extensive testing, neither of which were evaluated in the current study. However, in spite of these limitations, we believe that the current study provides a genetic basis for a novel testing scheme to supplement the cascade testing regimen for the efficient preventive medical care of pre-symptomatic BRs.

## Registry and the registration no. of the study/trial.

N/A.

## Animal studies

N/A.

## Supplementary Information

Below is the link to the electronic supplementary material.

Supplementary file1 (DOCX 272 KB)

Supplementary file2 (XLSX 32 KB)

Supplementary file3 (XLSX 91 KB)

## Data Availability

Germline SNV/indel data will be submitted to ClinVar by the time of acceptance of the manuscript under the following ClinVar submission accession IDs: SCV005045649—SCV005045664. The SUB14473776 ClinVar submissions can also be accessed here: https://www.ncbi.nlm.nih.gov/clinvar/submitters/507256/
