# Supplementary material for: High-risk pathogenic germline variants in blood relatives of BRCA1/2 negative probands
Source: Breast Cancer. 2024 Jul 13;31(6):1028–36. doi: 10.1007/s12282-024-01615-0 (PMC11489291; doi:10.1007/s12282-024-01615-0)
Supplement: Supplementary file 1 — Supplementary file1 (DOCX 272 KB) [file 12282_2024_1615_MOESM1_ESM.docx]

**Supplementary Methods**

**Subjects overlap between previous and current studies**

As research subjects, 13 probands and 34 blood relatives (BRs) overlapped between this and our previous study [1].

**Sample preparation for sequencing**

DNA from whole blood and saliva was extracted using the QIAamp DNA Blood Mini Kit (Qiagen) and Oragene DNA Kit (DNA Genotek). DNA quality and quantity were checked with a NanoDrop 2000 (Thermo Fisher Scientific) and Qubit 2.0 fluorometer (Thermo Fisher Scientific). DNA samples that passed the criteria for DNA purity (optical density 260/280 nm >1.8), ratio of dsDNA/ ssDNA concentration (>0.35), and dsDNA concentration (>50 ng/μl) were further processed to exome or panel sequencing.

**Library preparation and target-panel or exome sequencing**

The methods used for exome and target-panel sequencing are described previously [1]. For capture sequencing, we used two exome kits (SureSelect Human All Exon V5 [50.39 Mbps] and SureSelectXT Human All Exon V5 + lncRNA [61.94 Mbps] [Agilent Technologies]) and three target panels (SureSelectXT Custom; Target Panel 1; 119 genes and 0.49 Mbps, Target Panel 2; 119 genes and 0.54 Mbps, and Target Panel 3; 785 genes and 3.31 Mbps [Agilent Technologies]) (Supplementary Table 1). These target regions covered all exons of the previously known 30 HBOC causative genes (see also “Gene selection and risk assignment” in Methods). The list of genes can be found in the following section below. The median coverage was 143, 166.5, 452, 360 and 129 reads for Human All Exon V5, Human All Exon V5 + lncRNA, Target Panel 1, Target Panel 2 and Target Panel 3 libraries, respectively. A total of 281 proband and 684 BR/spouse germline specimens finally passed the stringent quality assessments during sample preparation, sequencing, and informatics analyses for targeted re-sequencing or exome sequencing.

**Gene selection and risk assignment**

In the current study, we focused on 30 previously known HBOC causative genes [1]. According to the NCCN guidelines for genetic/familial high-risk assessment, breast, ovarian, and pancreatic (2022 v2) [2] genes were divided into 5 risk classes based on their lifetime risk: high-risk genes (*BRCA1*, *BRCA2* and *TP53*); level-1 moderate-risk genes (*STK11*, *PTEN*, *CDH1* and *PALB2*), level-2 moderate-risk genes (*ATM*, *BARD1*, *CHEK2*, *NF1*, *RAD51C* and *RAD51D*)*,* low-risk genes (*EPCAM*, *MLH1*, *MSH2*, *MSH6* and *PMS2*), and genes with insufficient evidence (*BRIP1*, *NBN*, *RAD50*, *XRCC2*, *RAD51B*, *MRE11*, *FANCC*, *BLM****,*** *FAM175A*, *RINT1, FANCM* and *RECQL*). Among these genes, PGVs were detected on *BRCA1*, *BRCA2*, *TP53*, *PALB2*, *ATM*, *CHEK2*, *BARD1*, *NF1*, *RAD51C*, *RAD51D*, *MLH1*, *MSH6*, *BLM*, *BRIP1*, *FANCM* and *MRE11* genes in the proband or BR samples.

**Germline variant analysis**

Sequenced reads were aligned with BWA (Burrows-Wheeler Aligner; ver. 0.6.1) to the reference human genome (hg19) [3]. GATK (GenomeAnalysisTK; ver. 3.4–46) was used to recalibrate variant quality scores and to perform local realignment [4]. Germline variants were called with GATK UnifiedGenotyper, GATK HaplotypeCaller (GATK ver. 3.4–0) [5], and DeepVariant (ver. 1.0) [6], and were considered genuine when detected by two of the three software. The detailed filters used to identify significant germline SNVs and indels are described elsewhere [1]. Germline CNVs were detected with the eXome-Hidden Markov Model (XHMM; ver. 1.0) [7]. The pathogenicity of a variant was interpreted according to ACMG-AMP guidelines [8, 9] for the 30 genes (see above in ‘Gene selection and risk assignment’) except for *TP53*, as previously described [1, 10]. For classification of the pathogenicity of *TP53*, we used ‘ClinGen *TP53* Expert Panel Specifications to the ACMG/AMP Variant Interpretation Guidelines Version 1.2’ [11, 12].

**Population databases and disease variant databases**

Minor allele frequencies in our cohort were compared with those from population databases for Japanese people; specifically, HGVD (Human Genetic Variation Database; ver. 2.30) [13] or TMM (Tohoku Medical Megabank Project; 14KJPN) [14, 15]. HGVD (*n* = 1,210) and TMM (*n* = 14,129) comprise only Japanese persons without major diseases, including cancer [13-15]. A non-cancer subset of “East-Asian” gnomAD (ver. 2.1.1) (*n* = 8,846) [16] was also used as a control (*n* = 24,185 in total). Reported interpretations and some additional information, such as reference literature for known variants, were obtained through the HGMD (ver. 2021.2) and ClinVar (5/Aug/2021).

**References**

1. Kaneyasu T, Mori S, Yamauchi H, Ohsumi S, Ohno S, Aoki D, et al. Prevalence of disease-causing genes in Japanese patients with BRCA1/2-wildtype hereditary breast and ovarian cancer syndrome. NPJ Breast Cancer. 2020; 6: 25.

2. National Comprehensive Cancer Network. NCCN Clinical Practice Guidelines in Oncology for Genetic/Familial High-Risk Assessment: Breast, Ovarian, and Pancreatic (Version 2.2022).

3. Li H, Durbin R. Fast and accurate short read alignment with Burrows-Wheeler transform. Bioinformatics. 2009; 25: 1754-60.

4. DePristo MA, Banks E, Poplin R, Garimella KV, Maguire JR, Hartl C, et al. A framework for variation discovery and genotyping using next-generation DNA sequencing data. Nat Genet. 2011; 43: 491-8.

5. Van der Auwera GA, Carneiro MO, Hartl C, Poplin R, Del Angel G, Levy-Moonshine A, et al. From FastQ data to high confidence variant calls: the Genome Analysis Toolkit best practices pipeline. Curr Protoc Bioinformatics. 2013; 43: 11 0 1- 0 33.

6. Poplin R, Chang PC, Alexander D, Schwartz S, Colthurst T, Ku A, et al. A universal SNP and small-indel variant caller using deep neural networks. Nat Biotechnol. 2018; 36: 983-7.

7. Fromer M, Moran JL, Chambert K, Banks E, Bergen SE, Ruderfer DM, et al. Discovery and statistical genotyping of copy-number variation from whole-exome sequencing depth. Am J Hum Genet. 2012; 91: 597-607.

8. Richards S, Aziz N, Bale S, Bick D, Das S, Gastier-Foster J, et al. Standards and guidelines for the interpretation of sequence variants: a joint consensus recommendation of the American College of Medical Genetics and Genomics and the Association for Molecular Pathology. Genet Med. 2015; 17: 405-24.

9. Maxwell KN, Hart SN, Vijai J, Schrader KA, Slavin TP, Thomas T, et al. Evaluation of ACMG-Guideline-Based Variant Classification of Cancer Susceptibility and Non-Cancer-Associated Genes in Families Affected by Breast Cancer. Am J Hum Genet. 2016; 98: 801-17.

10. Yoshida R, Hagio T, Kaneyasu T, Gotoh O, Osako T, Tanaka N, et al. Pathogenicity assessment of variants for breast cancer susceptibility genes based on BRCAness of tumor sample. Cancer Sci. 2021; 112: 1310-9.

11. Fortuno C, Lee K, Olivier M, Pesaran T, Mai PL, de Andrade KC, et al. Specifications of the ACMG/AMP variant interpretation guidelines for germline TP53 variants. Hum Mutat. 2021; 42: 223-36.

12. ClinGen *TP53* Expert Panel Specifications to the ACMG/AMP Variant Interpretation Guidelines Version 1.2. <https://clinicalgenome.org/affiliation/50013/>. Accessed 23 May 2024

13. Higasa K, Miyake N, Yoshimura J, Okamura K, Niihori T, Saitsu H, et al. Human genetic variation database, a reference database of genetic variations in the Japanese population. J Hum Genet. 2016; 61: 547-53.

14. Nagasaki M, Yasuda J, Katsuoka F, Nariai N, Kojima K, Kawai Y, et al. Rare variant discovery by deep whole-genome sequencing of 1,070 Japanese individuals. Nat Commun. 2015; 6: 8018.

15. Yamaguchi-Kabata Y, Nariai N, Kawai Y, Sato Y, Kojima K, Tateno M, et al. iJGVD: an integrative Japanese genome variation database based on whole-genome sequencing. Hum Genome Var. 2015; 2: 15050.

16. Karczewski KJ, Francioli LC, Tiao G, Cummings BB, Alfoldi J, Wang Q, et al. The mutational constraint spectrum quantified from variation in 141,456 humans. Nature. 2020; 581: 434-43.

**Table S1.** Selection bias among the participating family members.

A. Participation rate in family number

|  | With Participant | Without Participant | % Participation |
| --- | --- | --- | --- |
| Number of Families | 281 | 742 | 27.5 |

B. Participation rate per characteristics of family members for families with participants

| Relation | | Participant | Non-participant | % Participation | Fisher Exact Test |
| --- | --- | --- | --- | --- | --- |
|  | BR | 682 | 3493 | 16.3 | *p* = 4.738 x 10^-11^  OR = 16.98 (4.482–96.52) |
|  | Spouse | 2 | 174 | 1.1 |  |
| BR: Gender | | | | | |
|  | Female | 494 | 1649 | 23.1 | Female vs Male  *p* = 2.686 x 10^-30^  OR = 2.753 (2.295–3.314) |
|  | Male | 188 | 1728 | 9.8 |  |
|  | Unknown | 0 | 116 | 0.0 |  |
| BR: Relatedness in Degree | | | | | |
|  | First | 497 | 492 | 50.3 | First vs Second or More  *p* = 6.822 x10^-205^  OR = 16.37 (13.48–19.94) |
|  | Second | 114 | 1417 | 7.4 |  |
|  | Third or More | 71 | 1584 | 4.3 |  |

*P*-values and odds ratio were computed by Fisher exact tests using allele counts.

Abbreviations: BR; blood relative, OR; odds ratio.

Odds ratio is shown with 95% confidence interval.

**Table S4.** Enrichment of pathogenic germline variant alleles in blood relatives compared with a non-cancer East-Asian population: Gene enrichment or depletion.

| Gene | Risk Class | Number of  PGV-positive BR^*^ | *P*-value | Odds Ratio  (95% Confidence Interval) |
| --- | --- | --- | --- | --- |
| *BRCA2* | High | 6 | 0.0135 | 3.2736 (1.3774-7.5208) |
| *BRCA1* | High | 3 | 0.2211 | 1.8962 (0.5020-5.8573) |
| *TP53* | High | 2 | 0.0190 | 11.7999 (1.7206-61.8147) |
| *PALB2* | Moderate 1 | 1 | 0.5789 | 1.1787 (0.0585-7.3436) |
| *RAD51D* | Moderate 2 | 4 | 0.0514 | 3.0149 (0.9905-8.0353) |
| *CHEK2* | Moderate 2 | 3 | 0.0615 | 3.5412 (0.9159-11.1820) |
| *RAD51C* | Moderate 2 | 2 | 0.1309 | 3.3707 (0.5631-13.6788) |
| *NF1* | Moderate 2 | 1 | 0.1994 | 0.2383 (0.0121-1.3163) |
| *ATM* | Moderate 2 | 0 | 0.1771 | 0.0431 (0.0000-1.6979) |
| *BARD1* | Moderate 2 | 0 | 1.0000 | 0.2068 (0.0000-8.642) |
| *MLH1* | Low | 0 | 0.0245 | 0.0224 (0.0000-0.8611) |
| *MSH6* | Low | 1 | 0.3776 | 2.2108 (0.1068-14.3022) |
| *FANCM* | Insufficient | 5 | 0.0004 | 9.3340 (3.328-25.2694) |
| *BRIP1* | Insufficient | 3 | 0.1325 | 2.4699 (0.6486-7.8755) |
| *BLM* | Insufficient | 2 | 0.6550 | 1.4149 (0.2447-5.3821) |
| *MRE11* | Insufficient | 4 | 0.0111 | 5.0623 (1.623-13.7972) |

^*^: One BR had two PGVs on *BRIP1* and *MRE11*.

*P*-values and odds ratio were computed by Fisher exact tests using allele counts. Red font is used to indicate significant enrichment or depletion in BRs in the current cohort. Abbreviations: BR; blood relative and PGV; pathogenic germline variant.

**Table S5.** Enrichment of pathogenic germline variant alleles in blood relatives compared with a non-cancer East-Asian population: Enrichment or depletion per risk class of gene.

| Risk Class | Number of  PGV-positive BR^*^ | *P*-value | Odds Ratio  (95% Confidence Interval) |
| --- | --- | --- | --- |
| High | 11 | 0.0016 | 3.0791 (1.5521-5.6694) |
| Moderate 1 | 1 | 0.5789 | 1.1787 (0.0585-7.3436) |
| Moderate 2 | 10 | 0.8703 | 1.0251 (0.5392-1.9364) |
| Low | 1 | 0.0986 | 0.2026 (0.0103-1.2015) |
| Insufficient | 14 | 0.0001 | 3.5618 (2.0378-6.2506) |

^*^: One BR had two PGVs on *BRIP1* and *MRE11* of the class of insufficient data.

Gene risk class assignment was determined as described in “Gene selection and risk assignment” in the Supplementary Methods. *P*-values and odds ratio were computed by Fisher exact tests using allele counts. Red font is used to indicate significant enrichment or depletion in BRs in the current cohort. Abbreviations: BR; blood relative and PGV; pathogenic germline variant.

**Supplementary Figures**

**Figure S1.** Representative pedigree charts of families in which a proband or a blood relative has any pathogenic germline variant.

Color codes of family ID indicate concordance or discordance of PGVs between probands and BRs. Females and males are represented as circles and squares, respectively. A numeric in a circle indicates the number of females omitted from the chart for simplicity. A diagonal line through the shape indicates a deceased person at the time the pedigree chart was drawn. Bold and narrow arrows indicate probands and BRs, respectively, who underwent genetic testing. The age below indicates either the age of onset of cancer where a cancer history was present, or the age of the member following receipt of a genetic test for the variant. Colors show affected cancer types. Genetic information is presented beneath each BR to describe when the member received a genetic test for the variant. Red and sky-blue fonts indicate mutant and wildtype alleles for the variant, respectively. Note: the pedigree chart of the A0281 family was previously presented [1] with genetic findings of the proband A0281 and her older sister (A0838); the data for the proband’s mother is new (A0873) and justifies its inclusion. Abbreviations: PGV; pathogenic germline variant, P; proband, BR; blood relative, Br; breast cancer, Pr; prostate cancer, U; uterine cancer, Li; liver cancer, CR; colorectal cancer, Ga; gastric cancer, and ML; malignant lymphoma.

**Figure S2.** Pedigree charts of families with confirmed and assumed *de novo* pathogenic germline variants of the *TP53* gene.

An orange family ID indicates discordance in the PGVs between the proband and the BRs/spouses. Females and males are represented as circles and squares, respectively. A diagonal line through the shape indicates a deceased person at the time the pedigree chart was drawn. Bold and narrow arrows indicate probands and BRs/spouses who received genetic testing. The age at the bottom indicates the age of onset of cancer if the BR/spouse had cancer history, the age when the BR/spouse received a genetic test for the variant, or the age of the BR/spouse when information for the BR/spouse was obtained from the proband. *TP53* variant (c. 713G>A [p.C238Y]) was found (BR, A0815) and assumed to be *de novo* because the proband (her mother; A0235) and the BR’s sister (A0812) lacked the variant, and her father had no previous history of cancer (nor was he subjected to testing in this instance). Colors show types of cancer. Genetic information is presented beneath each BR/spouse where the member received a genetic test for the variant. Red and sky-blue fonts are used to indicate the mutant and wildtype alleles for the variant, respectively. Abbreviations: PGV; pathogenic germline variant, P; proband, BR; blood relative, Br; breast cancer, Pa; pancreas, cancer, Ov; ovarian cancer, CR; colorectal cancer, Ga; gastric cancer, Le; leukemia, and BT; brain tumor.

**Supplementary Figure 3.** Route of pathogenic germline variant inheritance inferred based on phenotype and uncovered by genotype.

We show the frequency of route of pathogenic germline variant (PGV) inheritance as maternal, paternal, or undetermined transmission. The proportion of families is indicated using stacked bar plots. Route of PGV inheritance was estimated based on phenotype (status of presence or absence for history of breast or ovarian cancer) or uncovered by genotype (PGV carrying status). The Sankey diagram is used to connect the inheritance route derived from the phenotype and that from genotype per family.

**Supplementary Document**

**Selection bias**

The characteristics of family members are available only for the families with participants but not for those without any participant due to the initial study design, in which we collected the information for all family members only from the former families. To estimate overall rate of participation, we consider that the number of families can be utilized as surrogate measure of number of subjects. Here from 281 of 1,023 (27.5%) families, 684 family members participated in the study (Figure 1 and Table S1A).

We evaluated the presence or absence of selection bias using the demographics of the family members in the families with participants (Table S1B). Among total 4,355 family members (4,178 BR and 177 spouses) possibly eligible in the 281 families, 684 family members (682 [16.3%] BRs and 2 [1.1%] spouses) participated in the study. Females (494 of 2,135 [23.1%) participated more than males (188 of 1,918 [9.8%]). In relatedness of the BR to the proband, the participation rates were 497 of 987 (50.4%), 114 of 1,535 (7.4%) and 71 of 1,656 (4.3%) for the first-degree, the second-degree, and the third-degree or more BRs (Table S1B). Using Fisher exact tests, we observed statistically significant more participation in the comparisons of BRs vs spouses (*p* = 4.959 x 10^-11^, odds ratio [OR] = 17.065 [4.504–96.991; 95% confidence interval]), females vs males (*p* = 1.053 x 10^-30^, OR = 2.770 [2.309–3.334]), and the first-degree BRs vs the second-degree or more BRs (*p* = 1.332 x10^-205^, OR = 16.463 [13.552–20.056]) (Table S1B). As such, some of the characteristics of the family members were significantly associated with their biased participation.

**Genetic findings of representative families**

Representative pedigree charts of families with a proband or BR having any PGV are shown in Supplementary Figures 1 and 2. Detailed information for BRs/spouses is available in Supplementary Table 2.

The A0139 family is presented as an example of a PGV-concordant family. The proband A0139 and her daughter contained the same PGV on *PALB2* p.R1086*. Genetic testing confirmed that the proband’s husband and siblings were all negative for any PGV on the HBOC-causative genes, including *PALB2*. In this family, the PGV-carrying status coincided with breast cancer history (Supplementary Figure 1).

We show B0215 as a representative family exhibiting discordance with different PGVs between the proband and BRs. Whereas proband B0215 contained *PALB2* p.R1086*, *BRCA2* p.I1859Kfs*3 was instead detected in her sister who had no previous history of cancer (Supplementary Figure 1).

The E0321 family is presented as an example of families for which PGVs were detected only in the BRs and not the proband. In the E0321 family, the proband with breast cancer was negative for *BRCA2* p. K2217*; her brother, without a history of cancer, was positive for the variant (Supplementary Figure 1).

The A0281 family included both concordant and discordant PGVs; whereas the proband A0281 had the *BRIP1* p.R356* mutation, the *MRE11* p.N511Yfs*2 variation was detected in the proband’s mother and sister. The mother also had the *BRIP1* p.R356* mutation (Supplementary Figure 1).

We found one confirmed and another assumed *de novo* PGV for the *TP53* gene in the cohort. A Trio analysis was performed on the A0350 family, which revealed *TP53* p.R248Q as a *de novo* variant in the daughter (with histories of breast, ovarian and pancreatic cancer) of the proband with breast cancer. Likewise, in the A0235 family, the proband with breast cancer had wildtype *TP53* whereas one of her daughters had the *TP53* p.C238Y mutation and had suffered from leukemia, breast cancer, and a brain tumor. The other daughter had wildtype *TP53*. The husband of the proband lacked any cancer history but did not undertake *TP53* genotyping. We therefore assume that this *TP53* p.C238Y mutation of the daughter arose as a *de novo* mutation (Supplementary Figure 2).
